# Work of Art, Art of Work: Artistic Literacy and Quality in Long-Term Dementia Care

**DOI:** 10.1093/geroni/igaa057.303

**Published:** 2020-12-16

**Authors:** Katie Aubrecht

**Affiliations:** St. Francis Xavier University, Antigonish, Nova Scotia, Canada

## Abstract

This paper shares results from a thematic analysis (Braun & Clarke, 2006) of semi-structured interviews with a purposive snowball sample of 15 leaders in dementia arts education and praxis from Canada, the United States and United Kingdom. Interviews were conducted as part of a multi-phased collaborative, interdisciplinary arts-informed research project that aimed to operationalize quality mental health and dementia care in long-term care (LTC) from a relational perspective, with a focus on LTC staff literacy. Artistic literacy that is cultivated through creative arts-making and public exhibiting was described by participants as crucial to supporting and promoting quality within long-term care. Quality was imagined as a work of art and operationalized in terms of artist competencies, capacities and conditions. Artists included LTC staff, residents and their family and friends. Our analysis identified five themes related to artistic literacy: space-making, validation, fostering community, means of engagement, vulnerability and resilience. Drawing on cultural sociology (Bourdieu, 1993, 1984) and aging studies theory (Basting, 2018), we consider and discuss the role of the arts in disrupting unexamined assumptions about quality in LTC and advancing innovation in LTC staff mental health and dementia care.

